# A potential association between tirzepatide and hypercalcemia in the setting of chronic hydrochlorothiazide use

**DOI:** 10.1530/EDM-25-0067

**Published:** 2025-09-05

**Authors:** Basil Nduma, Sai Nikhitha Malapati, Veeranna Vibhuti

**Affiliations:** ^1^ Department of Internal Medicine, Medical City Arlington, Arlington, Texas, USA; ^2^ Department of Internal Medicine, Kamineni Academy of Medical Sciences and Research Center, Hyderabad, Telangana, India

**Keywords:** tirzepatide, hydrochlorothiazide (HCTZ), chronic kidney disease (CKD), hypercalcemia, type 2 diabetes mellitus (T2DM)

## Abstract

**Summary:**

Hypercalcemia is a prevalent electrolyte disturbance commonly associated with primary hyperparathyroidism, cancer, or medication adverse effects. Thiazide diuretics reduce urinary calcium excretion, increasing calcium reabsorption and hypercalcemia. Tirzepatide, a dual GIP and GLP-1 receptor agonist, is increasingly used for type 2 diabetes and obesity. While GIP/GLP-1 agonists typically have negligible effects on calcium homeostasis, the interaction between tirzepatide and thiazides remains unstudied. We report a 65-year-old female with obesity, hypertension, CKD3, and T2DM on chronic HCTZ who developed symptomatic hypercalcemia (corrected calcium: 4.58 mmol/L; normal range: 2.12–2.62 mmol/L), resulting in altered mental status days after initiating tirzepatide. PTH and vitamin D levels were low, and imaging ruled out malignancy. Discontinuation of tirzepatide/HCTZ, IV hydration, and calcitonin normalized her calcium by hospital day 4. This case highlights a potential association between HCTZ and tirzepatide in causing severe hypercalcemia. No prior reports link tirzepatide (or its combination with thiazides) to hypercalcemia. The mechanism likely involves thiazide-induced calcium reabsorption and tirzepatide’s effects on bone turnover. As the use of tirzepatide and other GLP-1/GIP agonists becomes more prevalent, clinicians need to closely monitor calcium levels in thiazide-treated individuals, particularly those with CKD. Additional research is also needed to elucidate the drug’s interaction with calcium metabolism.

**Learning points:**

- Clinicians should be aware of the potential for severe hypercalcemia when tirzepatide is co-administered with chronic thiazide diuretics, particularly hydrochlorothiazide (HCTZ), in patients with pre-existing CKD.
- Tirzepatide, a dual GIP and GLP-1 receptor agonist, may influence calcium metabolism through mechanisms including increased osteoblastic activity and altered PTH regulation, especially in individuals with impaired renal clearance.
- Baseline and follow-up serum calcium monitoring is strongly recommended within 1–2 weeks of initiating tirzepatide in patients receiving thiazide diuretics or those with CKD.
- This case suggests a possible drug–drug interaction between tirzepatide and HCTZ leading to symptomatic hypercalcemia, highlighting the need for pharmacovigilance as newer agents are integrated into routine diabetes care.
- Severe hypercalcemia can present with nonspecific symptoms such as altered mental status, fatigue, constipation, and polyuria; clinicians should maintain a high index of suspicion in susceptible populations.
- Prompt cessation of the suspected offending agents, hydration, and short-term use of calcitonin can result in rapid and sustained normalization of calcium levels without the need for bisphosphonates.

## Background

Hypercalcemia is a common electrolyte disturbance, often associated with primary hyperparathyroidism, malignancy, and medication effects (1). Thiazide diuretics, including hydrochlorothiazide (HCTZ), have long been recognized as contributors to hypercalcemia by reducing urinary calcium excretion (2). Other common causes of hypercalcemia such as granulomatous diseases (e.g., sarcoidosis), vitamin A toxicity, lithium use, and multiple myeloma were clinically excluded based on imaging, medication review, and absence of clinical features suggestive of these etiologies. Meanwhile, tirzepatide, a dual glucose-dependent insulinotropic polypeptide (GIP) and glucagon-like peptide-1 (GLP-1) receptor agonist, is an emerging therapeutic option for type 2 diabetes and obesity (3). While GLP-1 receptor agonists have been associated with minor calcium homeostasis disturbances (6), the interplay between tirzepatide and chronic thiazide use remains unknown. Here, we present a case in which a single dose of tirzepatide in a patient on chronic HCTZ therapy precipitated profound symptomatic hypercalcemia.

## Case presentation

A 65-year-old obese female with a medical history of hypertension (HTN), hyperlipidemia (HLD), hypothyroidism, chronic kidney disease 3 (CKD3), and diabetes mellitus type 2 (T2DM) presented to the emergency room (ER) with generalized weakness and altered mental status (AMS). She also experienced fatigue, mild constipation, and polyuria, consistent with hypercalcemia. These symptoms began 3 days after receiving her first-ever dose of tirzepatide (2.5 mg subcutaneously). Of note, the patient had been taking Losartan/HCTZ 100/25 mg daily for her hypertension for several years. She was not taking any calcium, vitamin D, over-the-counter supplements, or other medications associated with hypercalcemia. Before tirzepatide initiation, labs showed baseline corrected calcium of 2.30 mmol/L (normal: 2.12–2.62 mmol/L), parathyroid hormone (PTH) 4.76 pmol/L (normal: 1.05–6.89 pmol/L), 25-OH vitamin D 85 nmol/L (normal: >50 nmol/L), and serum creatinine 106 μmol/L (normal: 53–115 μmol/L). The patient was found by her mother on the floor, confused and minimally responsive. Emergency Medical Services (EMS) was called, and on arrival, EMS found the patient severely dehydrated. She received resuscitation fluid before being transported to the ER. Initial vital signs included respiratory rate of 20 breaths/min, pulse of 104 beats/min, temperature of 97.8°F (36.6°C), blood pressure of 220/93 mmHg, and oxygen saturation of 96%. Initial laboratory workup is presented in Tables 1 and 2.

**Table 1:** Laboratory results which were significant.

Laboratory test	Values	Normal range
Serum creatinine, μmol/L	256	53–115
Baseline	106	
Corrected calcium, mmol/L	4.58	2.12–2.62
Hemoglobin, g/L	140	120–160

**Table 2:** Laboratory results for PTH, TSH, free T4 (FT4), and vitamin D.

Test	Result
PTH intact, pg/mL	14.9 (low)
TSH, mU/L	0.16 (low)
FT4, ng/dL	1.40 (normal)
Vitamin D1,25, pg/mL	<5.0 (low)
Vitamin D25, ng/mL	17/7 (low)

On examination, the patient appeared lethargic but was responsive and coherent, with no focal neurological deficits, thyromegaly or lymphadenopathy. Cardiovascular, pulmonary, abdominal, and neurological exams were unremarkable. ECG showed normal sinus rhythm without QTc prolongation or arrhythmias.

Imaging was performed to rule out malignancy, metastatic disease, and other structural causes of altered mental status or hypercalcemia. CT of the chest, abdomen, pelvis, and MRI of the brain showed no acute abnormalities. The management strategy was directed by the current treatment guidelines, which included immediate cessation of tirzepatide and HCTZ and aggressive intravenous fluid hydration. Blood pressure was controlled with IV hydralazine and labetalol as needed and calcitonin injections were administered as an adjunct to aid in lowering the very high serum calcium level. Calcitonin was chosen due to its rapid onset of action, typically within minutes, compared to bisphosphonates, which require 48–72 h to take effect. Given the patient’s rapid clinical and biochemical improvement following calcitonin administration, discontinuation of the suspected offending agents, and aggressive intravenous hydration, we opted to avoid additional medications, favoring a more conservative approach once therapeutic goals were being met. The patient’s calcium level was measured daily and it normalized by day 4 of admission, and her blood pressure was managed with the addition of amlodipine.

Repeat labs at outpatient follow-up 3 weeks later showed sustained normocalcemia (corrected calcium: 2.45 mmol/L).

## Discussion

This article showcases a 65-year-old obese lady with a history of chronic kidney disease stage 3 (CKD3), type 2 diabetes mellitus (T2DM), and hypertension on HCTZ for several years, who developed severe hypercalcemia and altered mental status a few days following initiation of the first dose of tirzepatide. The timing of the onset of the events above suggests a possible synergistic effect of HCTZ and tirzepatide on calcium homeostasis, particularly in the setting of CKD. We considered dehydration as one of the causes of this patient’s hypercalcemia; however, it is very rare for dehydration alone to cause hypercalcemia with corrected calcium above 3.00 mmol/L. Thus, this article investigates other factors contributing to the severe hypercalcemia, including HCTZ use, tirzepatide use, and her history of chronic kidney disease. We also ruled out other causes of severe hypercalcemia as documented in our workup – including hypercalcemia of malignancy, primary hyperparathyroidism, granulomatous diseases, and thyrotoxicosis – which could typically present with corrected calcium levels above 3.50 mmol/L.

### Mechanism of thiazide-induced hypercalcemia

Hydrochlorothiazide (HCTZ) is a diuretic medication in the class of thiazide diuretics. This class of medication works by binding to the sodium-chloride symporter in the distal convoluted tubules. This inhibits sodium and chloride reabsorption in the distal convoluted tubules, leading to sodium and fluid loss. The inhibition of the sodium-chloride symporter increases the activity of the sodium-calcium exchanger, resulting in increased calcium reabsorption into the blood and resultant hypercalcemia (1). Furthermore, in CKD patients, chronic thiazide use can increase serum calcium levels by increasing distal convoluted tubular calcium absorption in these patients who already have problems with calcium excretion due to hyperphosphatemia and low calcitriol production, leading to secondary hyperparathyroidism, which further increases serum calcium levels (2). In addition, some genetic factors, such as calcium-sensing receptor polymorphisms, can predispose certain individuals to thiazide-induced hypercalcemia (3).

### Mechanism of GLP-1 and GIP on calcium homeostasis

Tirzepatide is both a glucose-dependent insulinotropic polypeptide (GIP) and glucagon-like peptide-1 (GLP-1) receptor agonist. Its effect on calcium metabolism is complex and not yet fully studied. The activation of GLP-1 receptors affects bone turnover by regulating osteoblast and osteoclast function and possibly reducing bone resorption (4). In animal studies, GLP-1 receptor agonists were shown to increase bone mineral density. However, their impact on serum calcium levels was unclear. On the other hand, studies have shown that GIP receptor agonists directly increase osteoblast activation along with an increase in calcium absorption in the intestine (5). The combination results in an enhancement in bone formation (5). Emerging data suggest dose-dependent effects of GIP on osteoblast activity and intestinal calcium absorption (5). While bone turnover markers were not available in this case, referenced studies report that both GIP and GLP-1 receptor agonists can stimulate parathyroid hormone (PTH) secretion in individuals with type 2 diabetes (6). This increase in PTH may transiently raise serum calcium levels by enhancing bone resorption (6, 7). Furthermore, in patients with chronic kidney disease who have reduced renal clearance, such elevations in serum calcium may be more sustained (2, 6, 7). The combined stimulation of osteoblastic activity and altered renal handling of calcium could provide a plausible explanation for the observed hypercalcemia in this patient.

In humans, GIP receptors are expressed in the parathyroid (PTH) gland, and this may suggest that there is a potential role of GIP activity in PTH regulation. A study by Kirsa *et al.* (6) found that both GIP and GLP-1 receptor agonism resulted in an increase in PTH secretion in individuals with diabetes, which could affect calcium homeostasis, resulting in increased serum calcium. Furthermore, the increased bone turnover from GIP receptor activation might also transiently increase serum calcium levels, especially in patients with compromised renal function who cannot efficiently excrete excess calcium (7).

### Strategies to prevent hypercalcemia in patients on HCTZ and tirzepatide

As noted in the mechanisms of these medications outlined above, both tirzepatide and HCTZ could potentially cause severe hypercalcemia, as seen in this patient. Some measures that could be taken to prevent this include close monitoring of patients who are started on GLP-1 and GIP agonists. Given that these are new medications on the market, they should be taken along with a continuum of care team who would be able to closely monitor the serum calcium levels of these patients and other adverse events, as well as provide an ongoing assessment of risk versus benefits (8). We recommend checking serum calcium within 1–2 weeks after initiating tirzepatide, especially in patients with CKD or those concurrently on thiazide diuretics.

Another strategy would be to discontinue thiazide diuretics in any patient who is at risk of hypercalcemia before starting GLP-1 and GIP agonists. A study by Teles *et al.*, recommended that patients with CKD stage 4 or stage 5 should be discontinued from thiazide medication and substituted with blood pressure medications with alternative mechanisms of action (9). The same study further emphasized that, per the 2018 ESC/ESH guideline for management of hypertension, thiazide diuretics should be avoided in patients with GFR 45 mL/min/1.73 m^2^, with loop diuretics preferred in these situations. In addition, measures such as significantly increased fluid intake and reduced calcium diets can help counter the retention of calcium.

Finally, pharmacological interventions could play a vital role in managing severe hypercalcemia. The gold standard is aggressive intravenous fluid hydration; however, other medications, such as calcitonin and bisphosphonates, are also being used as adjuncts. Denosumab (RANKL inhibitor) is also being used for hypercalcemia treatment in CKD patients, given that it does not require renal clearance (10).

## Conclusion

This case highlights a potential synergistic relationship between HCTZ and tirzepatide in causing severe hypercalcemia in a patient with CKD stage 3. An extensive literature review did not reveal any previous documentation of tirzepatide, or a combination of tirzepatide and HCTZ, as a cause of severe hypercalcemia. Since a single case report cannot establish causality, it is worth investigating further the effect of tirzepatide on calcium homeostasis, as well as the drug-drug interaction relationship between tirzepatide and HCTZ. Given the temporal relationship observed, further research is warranted to explore the potential effects of tirzepatide on calcium metabolism, particularly in the context of concurrent thiazide diuretic use. Furthermore, given the increasing use and high demand for tirzepatide and other GLP-1 agonists, further investigations and a better understanding of its effect on calcium homeostasis are warranted. Second, there should be close monitoring of calcium levels in patients starting GIP and GLP-1 agonists, especially in patient populations such as CKD patients or patients on HCTZ who are already susceptible to hypercalcemia.

## Declaration of interest

The authors declare that there is no conflict of interest that could be perceived as prejudicing the impartiality of the work reported.

## Funding

There was no other external source of funding for this report. This research was supported (in whole or in part) by HCA Healthcare and/or an HCA Healthcare-affiliated entity. The views expressed in this publication represent those of the author(s) and do not necessarily represent the official views of HCA Healthcare or any of its affiliated entities.

## Patient consent

Written informed consent has been obtained from the patient for the publication of anonymized clinical data.

## Author contribution statement

SNM conceived the idea of reporting this case and obtained the patient’s detailed history, and observed the clinical course of the patient. She was also present during the consent discussion alongside BN. SNM drafted the initial manuscript and led the revisions. BN was involved in the patient’s clinical care and collaborated in the consent process. He contributed significantly to the manuscript’s critical revisions and literature verification. VV supervised the case management and approved the reporting of the case. All authors reviewed and approved the final version of the manuscript.

## Ethical approval

This research activity was determined to be exempt or excluded from the HCA Healthcare Graduate Medical Education Institutional Review Board oversight in accordance with current regulations and institutional policy.
